# Factors associated with perceived weight gain among the adult population of Kazakhstan during the COVID-19 pandemic

**DOI:** 10.1371/journal.pone.0339619

**Published:** 2025-12-29

**Authors:** Assel Bukharbayeva, Alissa Davis, Susan L. Rosenthal, Akbope Myrkassymova, Balnur Iskakova, Aigulsum Izekenova, Assel Izekenova, Maral Yerdenova, Kuanysh Karibayev, Baurzhan Zhussupov, Gaukhar Mergenova

**Affiliations:** 1 Department of Epidemiology with a course of HIV-infection, Asfendiyarov Kazakh National Medical University, Almaty, Kazakhstan; 2 School of Social Work, Columbia University, New York, New York, United States of America; 3 Departments of Pediatrics and Psychiatry, Vagelos College of Physicians and Surgeons, Columbia University Irving Medical Center, New York, New York, United States of America; 4 Department of Biostatistics, Asfendiyarov Kazakh National Medical University, Almaty, Kazakhstan; 5 Department of Finance and Accounting, Kenzhegali Sagadiyev University of International Business, Almaty, Kazakhstan; 6 Global Health Research Center of Central Asia, Almaty, Kazakhstan; Mizan-Tepi University, ETHIOPIA

## Abstract

**Objective:**

Obesity has been recognized as a major public health problem globally. The COVID-19 pandemic has negatively affected behavioral patterns and, as a result, the weight gain of people around the world. We aimed to explore factors associated with perceived weight gain among the adult population of Kazakhstan during the COVID-19 pandemic.

**Methods:**

A cross-sectional study using a multi-stage sampling approach was conducted in Kazakhstan in the summer of 2021. We collected socio-demographic characteristics, information about health-related factors, behavioral risk factors, and self-reported weight change during the pandemic. Multivariable logistic regression was performed to identify factors related to weight gain during the pandemic. Data were analyzed using SAS 9.4 Software.

**Results:**

Among 931 participants, 20.95% (N = 195) of individuals gained weight. In the multivariable regression model, the weight gain was associated with increased snacking (AOR = 3.36, 95% CI: 2.20–5.12, p-value < .0001), decreased physical activity (AOR = 1.83, 95% CI: 1.24–2.70, p-value = 0.002), and increased alcohol consumption (AOR = 3.64, 95% CI: 1.72–7.70, p-value < 0.001). We found that older age (≥60 y.o.) was a protective factor for weight gain (AOR = 0.31, 95% CI: 0.15–0.62, p-value = 0.001).

**Conclusion:**

One-fifth of adults have gained weight during the COVID-19 pandemic which was associated with potentially modifiable unhealthy behavioral patterns. Our findings highlight that targeted health promotion strategies on global and national levels are needed to emphasize the avoidance of unhealthy behavioral patterns in times of crisis, like the COVID-19 pandemic.

## Introduction

The COVID-19 pandemic has been one of the biggest challenges for the entire world, forcing different countries to implement strategies such as strict quarantine measures, city- and country-wide lockdowns, vaccination campaigns, and more [1,2]. Kazakhstan has also taken the aforementioned anti-epidemiological measures, including the introduction of a state of emergency from March 16 to May 11, 2020, a two-stage relaxation of strict restrictive measures, which led to an increase in the incidence of novel coronavirus infection, and the need to strengthen restrictive measures from July 5, 2020 [3].

Disruptions of daily routines related to the COVID-19 lockdowns have impacted multiple behavioral patterns, including dietary habits, physical activity levels, intake of alcohol, and nutritional outcomes, which were affected globally [4]. In an online cross-sectional survey conducted during Poland’s nationwide lockdown, over 40.0% of adults reported increased food intake [1]. According to the study, overweight and obese people gained weight more often than others [1]. A study of United Kingdom adults found that during the COVID-19 lockdown, higher BMI was associated with lower levels of diet quality, and a greater reported frequency of overeating [5]. Moreover, self-reported decline in mental health because of the COVID-19 crisis was also predictive of greater overeating in lockdown [5]. The relevance of studying weight gain is based on the fact that a higher body mass index has been associated with poor outcomes in adult patients with COVID-19, i.e., people with obesity are more adversely affected by the COVID-19 virus compared to people with normal weight [6,7]. In a meta-analysis of the impact of increased BMI and obesity on novel coronavirus infection outcomes in adults, both higher BMI and obesity were associated with severe COVID-19, admission to the intensive care unit, acute respiratory distress syndrome, use of mechanical ventilation, and mortality in adult patients with COVID-19 [6]. According to the literature, predictors of weight gain during the COVID-19 pandemic in adults include several factors such as separated marital status, lower socioeconomic level, increased total food intake, decreased physical activity, younger age, female gender, decreased sleep quality, changes in eating behavior, and others [7]. Therefore, gaining a more precise understanding of how the lockdowns influenced nutritional and lifestyle behaviors is essential for developing and implementing public measures. These measures are crucial for safeguarding the health of diverse populations during potential future pandemics or natural disasters of various durations and levels. To our knowledge, there is limited research to assess weight gain among the adult population of Kazakhstan during the COVID-19 pandemic, and our study fills an important gap in the literature.

Therefore, we have aimed to study factors associated with perceived weight gain among the adult population of Kazakhstan during the COVID-19 pandemic.

## Materials and methods

### Study design

The study design was described previously elsewhere [8]. We conducted a cross-sectional face-to-face survey of 1,021 participants between June 26 and July 10, 2021. Data collection was performed by the Public Opinion Research Centre. Participants were recruited using a multi-stage sampling approach. Briefly, in the first stage, strata that represent the administrative region of the country were identified. The sampling plan was based on the administrative-territorial division of the Republic of Kazakhstan. Fourteen regions of Kazakhstan were considered as a separate stratum, and three cities of republican significance (Nur-Sultan, Almaty and Shymkent) were self-representative strata. Within each stratum, except for three cities of republican significance, two substrates were identified – urban and rural areas. In the second stage, the settlements where the research will be conducted were selected: the largest city in the administrative region and a randomly selected rural settlement. In the third stage, random route sampling was used to identify households for the survey. The survey was conducted from June 26 to July 10, 2021. Individuals who were at least 18 years old, living in Kazakhstan, and did not have a cognitive or severe psychiatric impairment that would prevent comprehension of study procedures, as assessed during the Informed Consent process, were eligible for the study. Exclusion criteria from the study included persons under the age of 18 years, persons under the influence of alcohol or drugs, and persons with cognitive or severe mental impairment that would interfere with understanding the study procedures, providing informed consent, and answering the questionnaire. Verbal informed consent was obtained from all participants of the study before the start of the survey. Verbal consent was documented by a research assistant during data collection in the electronic questionnaire as a “yes/no” option. If a person has given consent to participate in the survey, a research assistant indicated it at the beginning of the survey, and the algorithm allowed the survey to continue; if a participant has not given consent, the survey was terminated. No replacement was considered for individuals who refused the interview. After a quality check, 30 incomplete records were dropped from the study, resulting in a sample size of 991 adults.

### Measures

#### Dependent variable – Weight gain.

We asked respondents to report weight change during the pandemic (Lost/Gained/Maintained weight or Do not know). We created a dichotomous variable that reflected weight change with two categories: gained weight and did not gain weight (the latter category included those who Lost/Maintained weight). We dropped those who responded that they did not know about weight changes during the pandemic. The final sample consisted of 931 adults.

#### Independent variables.

The socio-demographic characteristics of the survey included self-reported gender, age, level of education (high and postgraduate, up to secondary or specialized secondary), employment status (full-time, part-time, unemployed or other), marital status (married, in civil marriage, in a long-term relationship, single, widowed, or divorced), and residency type (urban or rural). We also asked if they work remotely from home (yes/no/don’t work or study). Study participants were asked to report their current body weight and height in order to determine their body mass index (BMI) which was defined as a weight in kilograms divided by the square of height in meters [9]. The correspondence between the mass of a person and his height is determined according to the recommendations of the World Health Organization (WHO) [9].

**Economic vulnerabilities:** We asked participants questions about their change of financial status (Deteriorated/Has not changed/Improved/Don’t know) and working conditions (Deteriorated/Has not changed/Improved) during the pandemic. For analysis purposes, binary variables were created. We have combined those who have reported no change, improvement or do not know about their financial status and working conditions into a reference group. We also asked participants if they faced food insecurity (yes/no) during the pandemic.

*Self-reported COVID-19*: We asked participants if they suspected that they have had, or currently have, COVID-19.

**Mental Health – Anxiety and depression:** To measure the presence of depressive and anxiety symptoms within the past two weeks, we used the Patient Health Questionnaire-4 (PHQ-4), which is a composite four-item screening scale consisting of the two core criteria for depressive disorders and the two core criteria for generalized anxiety disorder [10]. The total composite score of PHQ–4 ranges from 0 to 12, and goes from normal (0–2) to mild (3–5) to moderate (6–8) to severe (9–12) [10].

*Sleep health – SATED*: Sleep health was assessed by the SATED questionnaire [11]. It is a self-administered questionnaire that is used to assess five key dimensions of sleep associated with health outcomes [11]. The five dimensions include Satisfaction with sleep, Alertness during waking hours, Timing of sleep, Sleep Efficiency, and Sleep Duration [11]. Each dimension is rated from 0 to 2, with 0 for “never/rarely,” 1 for “sometimes,” and 2 for “usually/always.” [11]. The total score ranges from 0 to 10 points. The higher the overall score, the higher the quality of sleep [11]. In our study, we considered 0 ≤ SATED ≤ 7 to be an indicator of poor sleep health, and SATED ≥ 8 was considered as good sleep health (reference group) [12].

*Chronic illness*: We asked participants to report any chronic illness that they have (liver disease, kidney disease, diabetes mellitus, hypertension, cardiovascular disease, asthma, chronic obstructive pulmonary disease, cancer, tuberculosis, HIV, mental illness, and others).

*Physical activity*: Study participants were asked about perceived changes in physical activity levels compared to before the COVID-19 pandemic (Increased/Decreased/Has not changed/Do not know). For analysis purposes, a binary variable was created (decreased physical activity compared to other physical activity statuses).

*Alcohol consumption*: We asked about perceived changes in the participants’ consumption of alcohol since the start of the COVID-19 pandemic.

*Eating behavior change – Snacking increase*: Study participants were asked about perceived changes in eating behavior, particularly in snacking (Decreased/Increased/Did not change/Not applicable).

### Statistical analysis

First, we computed descriptive statistics (i.e., means and standard deviations for continuous variables and frequencies and percentages for categorical variables) to describe the study population based on socio-demographic characteristics as well as health-related and lifestyle-related measures.

We conducted logistic regression analyses to examine which factors were associated with weight gain during the COVID-19 pandemic. Bivariate logistic regression analyses were conducted to identify potential associations with all factors we hypothesized to be associated with the dependent variable. All associated factors (p-value <0.1) with the dependent variable were included in the final model for multivariable logistic regression analyses. Adjusted odds ratios (AOR) with 95% confidence intervals (CI) were calculated to assess statistically significant associations between weight gain during the pandemic and independent predictor variables. Statistical significance for the final model was set at a p-value < 0.05. We checked variables for multicollinearity before including them in the final model. Data were analyzed using SAS 9.4 Software.

### Ethical approvals

The study was approved by the Ethical Committee No. 10 of the Asfendiyarov Kazakh National Medical University on September 30th, 2020.

## Results

The socio-demographic characteristics of the study population (N = 931), along with the variables included in the analysis, are summarized in Table 1. The mean age of participants was 41.24 (SD = 15.00) years old, the largest group was people aged 30–44 years, and about half of the sample were women (53.06%). Over a third of respondents experienced deterioration in their financial status (37.06%). Of the total number of respondents, 392 respondents (42.11%) noted that during the pandemic, they were worried that they would not have enough money to buy food for themselves and their loved ones. According to descriptive analysis, 195 (20.95%) adults reported perceived weight gain, and 478 (51.34%) were overweight (pre-obesity, obesity). The results demonstrated that 20% of respondents (N = 187) had decreased physical activity compared to the pre-pandemic period. Almost one-fifth of the respondents (N = 171, 18.37%) self-reported COVID-19, and 322 (34.59%) of them had a chronic illness. The study results showed that during the pandemic, approximately 13.53% (N = 126) of the total sample (N = 931) reported increased snacking (Table 1).

**Table 1:** Socio-demographic characteristics of the sample (N = 931).

Characteristics	Total sample	Gained weight 195 (20.95%)	Lost/Maintained weight 736 (79.05%)	p-value
	**Mean (SD)**			
Age (in years)	41.24 (15.00)	37.70 (12.70)	42.18 (15.43)	0.0002
PHQ-4 (total score)	1.60 (2.25)	1.98 (2.56)	1.50 (2.15)	0.007
	**N (%)**			
Age group				0.0002
18–29 y.o.	229 (24.60)	53 (5.69)	176 (18.90)	
30–44 y.o.	310 (33.30)	76 (8.16)	234 (25.13)	
45–59 y.o.	243 (26.10)	55 (5.91)	188 (20.19)	
≥ 60 y.o.	149 (16.00)	11 (1.18)	138 (14.82)	
Gender				0.043
Male	437 (46.94)	79 (8.49)	358 (38.45)	
Female	494 (53.06)	116 (12.46)	378 (40.60)	
Marital status				0.582
Married, in a civil marriage, in a long-term relationship	581 (62.41)	125 (13.43)	456 (48.98)	
Single, widowed, divorced	350 (37.59)	70 (7.52)	280 (30.08)	
Education				0.052
High and postgraduate	389 (41.78)	94 (10.10)	295 (31.69)	
Up to secondary	226 (24.27)	36 (3.87)	190 (20.41) \	
Specialized secondary	316 (33.94)	65 (6.98)	251 (26.96)	
Current employment status				0.694
Full-time	502 (53.92)	108 (11.60)	394 (42.32)	
Part-time	90 (9.67)	19 (2.04)	71 (7.63)	
Unemployed	73 (7.84)	18 (1.93)	55 (5.91)	
Other	266 (28.57)	50 (5.37)	216 (23.20)	
Area of residence				0.068
Rural	373 (40.06)	67 (7.20)	306 (32.87)	
Urban	558 (59.94)	128 (13.75)	430 (46.19)	
Financial status deteriorated (yes)	345 (37.06)	84 (9.02)	261 (28.03)	0.050
Food insecurity (yes)	392 (42.11)	97 (10.42)	295 (31.69)	0.015
Working remotely from home (yes, completely or partly)	140 (15.04)	30 (3.22)	110 (11.82)	0.879
Physical activity decreased (yes)	187 (20.09)	61 (6.55)	126 (13.53)	<.0001
Chronic illness (yes)	322 (34.59)	72 (7.73)	250 (26.85)	0.440
Self-reported COVID-19 (yes)	171 (18.37)	39 (4.19)	132 (14.18)	0.779
Poor Sleep Health (SATED < 8)	357 (38.35)	73 (7.84)	284 (30.50)	0.769
Alcohol consumption increase (yes)	35 (3.76)	20 (2.15)	15 (1.61)	<.0001
Increased snacking (yes)	126 (13.53)	59 (6.34)	67 (7.20)	<.0001
BMI category				<.0001
≥ 25 – Overweight (pre-obesity, obesity)	478 (51.34)	134 (14.39)	344 (36.95)	
< 25 - Normal weight, Underweight	453 (48.66)	61 (6.55)	392 (42.11)	
Moderate and severe mental health symptoms	64 (6.87)	19 (2.04)	45 (4.83)	0.075

*SD – standard deviation.

In the crude logistic regression model, we found that older age (≥60 y.o.) (OR:0.27; 95%CI[0.13–0.53], p-value < 0.001), lower level of education (OR:0.60, 95%CI [0.39–0.91], p-value = 0.017) were associated with higher odds of weight gain during the pandemic (Table 2). At the same time, being female (OR:1.39, 95%CI[1.01–1.92], p-value = 0.044), experiencing food insecurity (OR:1.48, 95%CI[1.08–2.03], p-value = 0.015), deterioration of financial status (OR:1.38, 95%CI[1.00–1.90], p-value = 0.051), having decreased physical activity (OR:2.20, 95%CI[1.54–3.15], p-value < .0001), having moderate and severe mental health symptoms (OR:1.09, 95%CI[1.02–1.17], p-value = 0.008), increased snacking (OR:4.33, 95%CI[2.92–6.43], p-value < .0001), and increased alcohol consumption (OR:5.49, 95%CI[2.76–10.95], p-value < .0001) were associated with weight gain during the COVID-19 pandemic (Table 2).

**Table 2:** Bivariate logistic regression estimates of odds ratios (OR) and 95% confidence intervals (CI) for the association between weight gain and studied variables.

Categorical Variables	Weight gain -	
	Bivariate Unadjusted OR [95% CI]	p-value
Age group (in years)		
18–29 y.o.	ref	
30–44 y.o.	1.08 [0.72 - 1.61]	0.712
45–59 y.o.	0.97 [0.63 - 1.49]	0.895
≥ 60 y.o.	0.27 [0.13 - 0.53]	< 0.001
Gender		
Male	Ref	
Female	1.39 [1.01 - 1.92]	0.044
Marital status		
Married, in relationships	Ref	
Single, widowed, divorced	0.91 [0.66 - 1.27]	0.582
Education		
Completed high or postgraduate degree	ref	
Up to secondary	0.60 [0.39 - 0.91]	0.017
Specialized secondary	0.81 [0.57 - 1.16]	0.256
Current employment status		
Full-time	ref	
Part-time	0.98 [0.56 - 1.69]	0.932
Unemployed	1.19 [0.67 - 2.12]	0.544
Other	0.84 [0.58 - 1.23]	0.376
Area of residence		
Rural	0.74 [0.53 - 1.02]	0.068
Urban	ref	
Had food insecurity (yes)	1.48 [1.08 - 2.03]	0.015
Physical activity decreased (yes)	2.20 [1.54 - 3.15]	<.0001
Financial status deteriorated (yes)	1.38 [1.00 - 1.90]	0.051
Worked remotely from home (yes)	1.04 [0.67 - 1.61]	0.878
Chronic illness (yes)	1.14 [0.82 - 1.58]	0.441
Self-reported COVID-19 (yes)	1.14 [0.77 - 1.70]	0.508
Moderate and severe mental health symptoms (yes)	1.09 [1.02 - 1.17]	0.008
Poor Sleep Health (SATED < 8)	1.05 [0.76 - 1.45]	0.769
Snacking increase	4.33 [2.92 - 6.43]	<.0001
Alcohol consumption increase	5.49 [2.76 - 10.95]	<.0001

**Fig 1:**
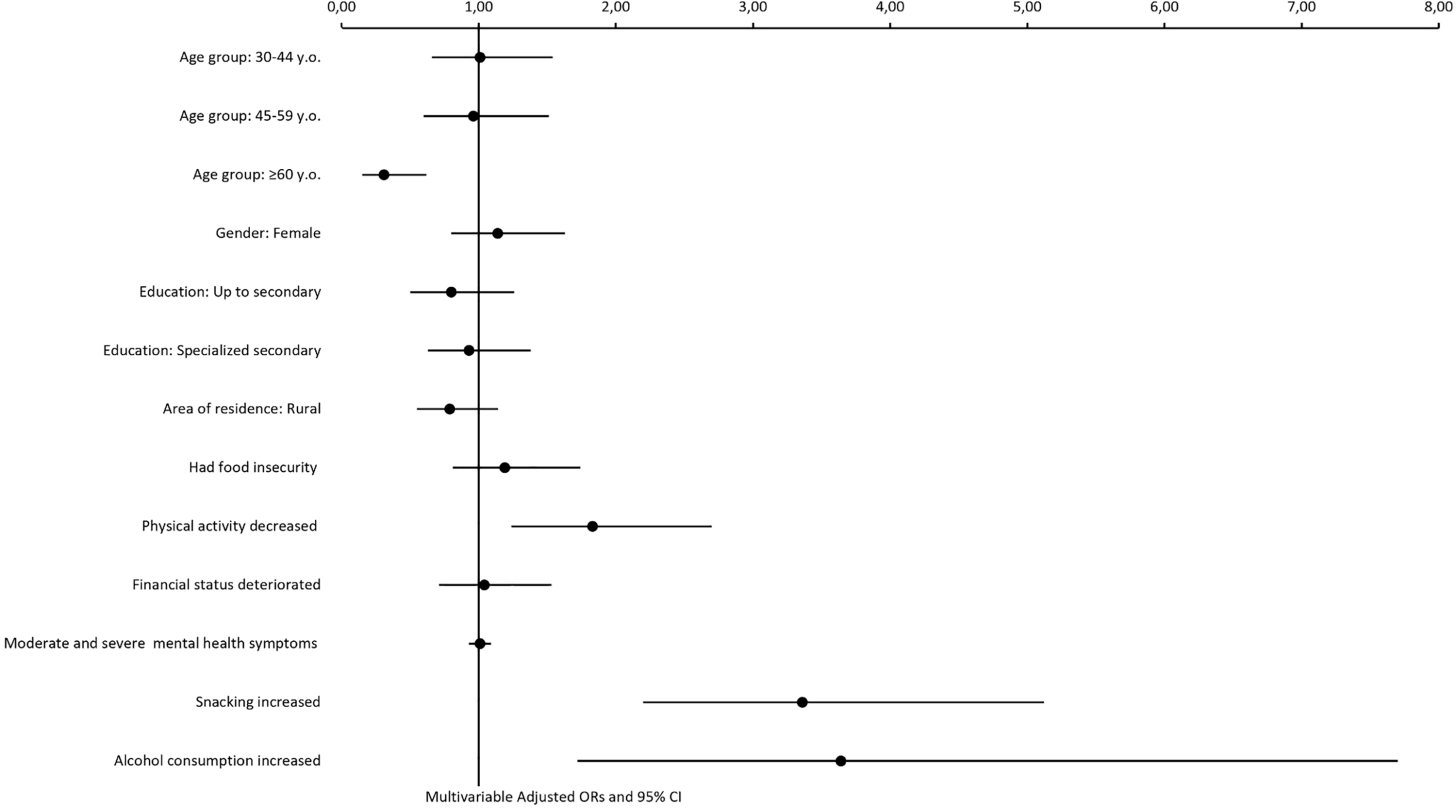
Forrest plot of multivariable adjusted odds ratios with 95% CI for weight gain and associated factors.

We performed a multivariable analysis (Table 3 and Fig 1) to examine the association between self-reported weight gain and multilevel factors that were found to be significantly (p-value <0.1) associated with the dependent variable in bivariate analyses. Increased snacking (AOR: 3.36, 95% CI[2.20–5.12], p-value < .0001), decreased physical activity (AOR:1.83, 95% CI[1.24–2.70], p-value = 0.002), and increased alcohol consumption (AOR: 3.64, 95% CI[1.72–7.70], p-value< 0.0010) were associated with higher odds of weight gain in multivariable model. In contrast, older individuals (≥60 y.o.) had 0.31 times the odds of weight gain compared to individuals who are younger than 60 years old (AOR: 0.31, 95% CI[0.15–0.62], p-value = 0.001). The Forrest plot of multivariable adjusted odds ratios for weight gain and associated factors is shown in Fig 1.

**Table 3:** Multivariable adjusted logistic regression estimates of odds ratios (OR) and 95% confidence intervals (CI) for the association between weight gain and studied variables*.

Categorical Variables	Weight gain	
	Multivariable Adjusted OR [95% CI]	p-value
Age group (in years)		
18–29 y.o.	ref	
30–44 y.o.	1.01 [0.66 - 1.54]	0.974
45–59 y.o.	0.96 [0.60 - 1.51]	0.842
≥ 60 y.o.	0.31 [0.15 - 0.62]	0.001
Gender		
Male	ref	
Female	1.14 [0.80 - 1.63]	0.465
Education		
Completed high or postgraduate degree	ref	
Up to secondary	0.80 [0.50 - 1.26]	0.328
Specialized secondary	0.93 [0.63 - 1.38]	0.727
Area of residence		
Rural	0.79 [0.55 - 1.14]	0.207
Urban	ref	
Had food insecurity (yes)	1.19 [0.81 - 1.74]	0.388
Physical activity decreased (yes)	1.83 [1.24 - 2.70]	0.002
Financial status deteriorated (yes)	1.04 [0.71 - 1.53]	0.844
Moderate and severe mental health symptoms	1.01 [0.93 - 1.09]	0.866
Snacking increase	3.36 [2.20 - 5.12]	<.0001
Alcohol consumption increase	3.64 [1.72 - 7.70]	< 0.001

*The Hosmer–Lemeshow Goodness-of-Fit test: p-value=0.651.

## Discussion

Even before the COVID-19 pandemic, obesity was a major public health problem globally [13]. According to the Global Nutrition Report about Kazakhstan, the prevalence of overweight and obesity in adults aged 18 years and over increased in recent years [14]. In the year 2000, 44.6% of women and 44.6% of men were overweight, and 15.8% of females and 11.7% of males were obese [14]. In 2016, more than 50% of males and females were overweight, and 18.9% of males and 22.7% of females had obesity [14]. Moreover, it has been projected that the prevalence of overweight and obesity will increase with time [14, 15]. This trend is worrying since obesity has emerged as an important risk factor for the exacerbation and mortality of COVID-19, alongside with increased risk for many serious diseases and health conditions. [16, 17, 18].

This study, conducted in a sample of 931 adults, provided an understanding of self-reported weight gain and food-related behavior during the COVID-19 pandemic in Kazakhstan. Our findings are consistent with the literature regarding variations of the effects of the pandemic on weight by age, physical activity, increased snacking, and increased alcohol consumption [19, 20, 7, 21].

Older age was a protective factor for weight gain in this study, which is consistent with the results of other studies [19,22]. In a study conducted by a group of researchers from China in 2023, participants belonging to a younger age group were found to be more vulnerable to weight gain [19]. In general, unhealthy eating behavior and associated weight gain during COVID-19 were common among young adults [22]. During the pandemic, one-third of young people gained weight [22]. According to a combined systematic review and meta-analysis, older adults (>60 years old) reported a significant loss in body weight during the COVID-19 lockdown [23]. Longitudinal study results suggest that young individuals were 3.34 times more likely to gain weight than older individuals [24]. In contrast, in a nationwide population-based study among American adults, those who reported weight gain were significantly more likely to be older (> 46 y.o.) [25].

Engaging in physical activity and exercise plays a pivotal role in upholding general health and wellness while also serving as a preventive measure against various chronic diseases, including cancer, obesity, and diabetes [26]. A decline in physical activity, coupled with increased calorie intake, leads to an increase in body weight [27]. Nutritional outcomes such as body mass index (BMI) are closely linked to both dietary and physical activity patterns [28].

A systematic review of 22 studies conducted in countries with different income levels revealed that COVID-19 lockdowns had a significant impact on the dietary and physical activity patterns of global populations [21]. This, in turn, led to changes in people’s body weight and BMI, with significant increases observed [21]. According to the review, the effect on physical activity was temporary, showing a decline during the lockdown period but a subsequent return to normal after the lockdown [21]. The authors suggest that the decrease in physical activity was likely driven by factors such as increased time spent at home, remote work arrangements, and increased screen time while at home, coupled with limited access to gyms [29, 30].

Increased alcohol consumption during the COVID-19 pandemic was linked with weight gain in our study population which is consistent with the existing literature [7,31, 32, 33]. According to a cross-sectional study conducted during the COVID-19 lockdown in Peru, increased alcohol consumption is a significant risk factor for weight gain [31]. In another cross-sectional study, weight gain was associated with increased alcohol consumption during the COVID-19 pandemic [32]. An online survey of Brazilian adults revealed that a higher frequency of alcoholic beverage intake as well as a dose of alcoholic beverage were predictors of weight gain [33].

In our study, participants reported increased snacking which is consistent with the literature [7, 33, 34]. It has been reported that the frequency of snacking between meals has increased during the pandemic compared to the pre-pandemic era [7, 34]. In a cross-sectional study among adults in Belgium, higher odds of weight gain were observed among those who increased the consumption of snacks [32]. In another study, predictors of weight gain included increased snacking and evening snacks during the pandemic period [33].

### Limitations

The current study had a few limitations. Firstly, as socio-demographic factors and all other characteristics of the study participants, including weight gain, were self-reported, there is the possibility of a recall bias and social desirability bias. Secondly, we have not distinguished healthy snacks from unhealthy ones. Thirdly, this study was limited by the cross-sectional design of the study, and therefore, no inferences about causal relationships can be made. Lastly, it is essential to consider the cultural context that influences self-perception of weight gain, as it can differ significantly from clinical weight gain [35].

## Conclusion

It is concerning that more than half of our study sample has excessive weight. Results of our study showed that several types of behavior were associated with weight gain during the COVID-19 pandemic among people in Kazakhstan. Data suggests that more research focused on behavioral risks of unhealthy weight gain and the development of guidelines for weight management for different age groups should be done in Kazakhstan. Health promotion strategies on a national level should be implemented to promote healthy eating, decrease alcohol consumption, increase physical activity, and adopt weight management/weight loss strategies among people in Kazakhstan.

## Supporting information

S1 Data Dataset. (CSV)
